# Presence of comorbidities alters management and worsens outcome of patients with acute respiratory distress syndrome: insights from the LUNG SAFE study

**DOI:** 10.1186/s13613-022-01015-7

**Published:** 2022-05-21

**Authors:** Emanuele Rezoagli, Bairbre A. McNicholas, Fabiana Madotto, Tài Pham, Giacomo Bellani, John G. Laffey, the LUNG SAFE Investigators, the ESICM Trials Group

**Affiliations:** 1 School of Medicine and Surgery, University of Milano-Bicocca, Monza, Italy; 2 Department of Emergency and Intensive Care, San Gerardo Hospital, Monza, Italy; 3 School of Medicine, National University of Ireland Galway, Galway, Ireland; 4 Dept of Anaesthesia and Intensive Care Medicine, Galway University Hospitals, Galway, Ireland; 5 Value based healthcare unit, IRCCS MultiMedica, Sesto San Giovanni, Milan, Italy; 6 Service de Médecine Intensive-Réanimation, AP-HP, Hôpital de Bicêtre, DMU 4 CORREVE Maladies du Cœur et Des Vaisseaux, FHU Sepsis, Groupe de Recherche Clinique CARMAS, Le Kremlin-Bicêtre, France; 7 Université Paris-Saclay, UVSQ, Univ. Paris-Sud, Inserm U1018, Equipe d’Epidémiologie respiratoire intégrative, CESP, 94807 Villejuif, France; 8 Lung Biology Group, Regenerative Medicine Institute (REMEDI) at CÚRAM Centre for Research in Medical Devices, Biomedical Sciences Building, National University of Ireland Galway, Galway, Ireland

**Keywords:** Comorbidities, Chronic respiratory impairment, Chronic renal failure, Chronic liver failure, Immune suppression, Congestive heart failure, Diabetes

## Abstract

**Background:**

The impact of underlying comorbidities on the clinical presentation, management and outcomes in patients with ARDS is poorly understood and deserves further investigation.

**Objectives:**

We examined these issue in patients with ARDS enrolled in the Large observational study to UNderstand the Global impact of Severe Acute respiratory FailurE (LUNG SAFE) study.

**Methods:**

In this secondary analysis of the patient cohort enrolled in the LUNG SAFE study, our primary objective was to determine the frequency, and impact of comorbidities on the management and ICU survival of patients with ARDS. Secondary outcomes relating to comorbidities included their impact on ventilatory management, the development of organ failures, and on end-of-life care.

**Results:**

Of 2813 patients in the study population, 1692 (60%) had 1 or more comorbidities, of whom 631 (22.4%) had chronic respiratory impairment, 290 (10.3%) had congestive heart failure, 286 (10.2%) had chronic renal failure, 112 (4%) had chronic liver failure, 584 (20.8%) had immune incompetence, and 613 (21.8%) had diabetes. Multiple comorbidities were frequently present, with 423 (25%) having 2 and 182 (11%) having at least 3 or more comorbidities. The use of invasive ventilation (1379 versus 998, 82 versus 89%), neuromuscular blockade (301 versus 249, 18 versus 22%), prone positioning (97 versus 104, 6 versus 9%) and ECMO (32 versus 46, 2 versus 4%) were each significantly reduced in patients with comorbidities as compared to patients with no comorbidity (1692 versus 1121, 60 versus 40%). ICU mortality increased from 27% (*n* = 303) in patients with no comorbidity to 39% (*n* = 661) in patients with any comorbidity. Congestive heart failure, chronic liver failure and immune incompetence were each independently associated with increased ICU mortality. Chronic liver failure and immune incompetence were independently associated with more decisions to limitation of life supporting measures.

**Conclusions:**

Most patients with ARDS have significant comorbidities, they receive less aggressive care, and have worse outcomes. Enhancing the care of these patients must be a priority for future clinical studies.

*Trial registration* LUNG-SAFE is registered with ClinicalTrials.gov, number NCT02010073.

**Supplementary Information:**

The online version contains supplementary material available at 10.1186/s13613-022-01015-7.

## Background

The impact of underlying comorbidities on the clinical presentation, management and outcomes in patients with ARDS is poorly understood and deserves further investigation. This knowledge gap is exacerbated by the fact that the evidence base for management of ARDS comes from clinical trials that frequently exclude these patients [1]. These trials have led to important clinical advances, including the recognition of the protective effects of lowered tidal volume [2], the role of prone positioning [3] and of muscle relaxants in early moderate–severe ARDS [4] among others.

A concern that has arisen is the generalizability of the findings of studies carried out in patients with no comorbidities, to the patient population with comorbidities, and the validity of these trials if only performed in a minority of the entire ARDS patient cohort. Furthermore, these concerns may limit the degree to which these interventions are applied to patients with significant underlying comorbidities [5].

Given the potential for important impact of comorbidities on the management and outcomes from ARDS, we wished to address these issues in this secondary analysis of the LUNG SAFE patient cohort. Briefly, LUNG SAFE was an international, multicentre, prospective cohort study of patients undergoing invasive or non-invasive ventilation, conducted during 4 consecutive weeks in the winter of 2014 in a convenience sample of 459 ICUs from 50 countries across 5 continents, that recruited 3,022 patients that fulfilled ARDS criteria [6].

Our primary objective, in this secondary LUNG SAFE analysis, was to determine the frequency, and impact of comorbidities on the management and ICU survival of patients with ARDS. Secondary outcomes included determination of the impact of these comorbidities on ventilatory management, the development of organ failures, and on end-of-life care.

## Materials and methods

This is a sub-study of the LUNG-SAFE study, an international, multicenter, prospective cohort study of patients receiving invasive or non-invasive ventilation, and the detailed methods and protocol have been published elsewhere [6]. The study, led by the European Society of Intensive Care Medicine (ESICM), was endorsed by multiple national societies/networks (Additional file 1: Appendix S1). All participating ICUs obtained ethics committee approval, and either patient consent or ethics committee waiver of consent. National coordinators and site investigators (Additional file 1: Appendix S1) were responsible for obtaining ethics committee approval and for ensuring data integrity and validity.

### Patients, study design and data collection

**Fig. 1:**
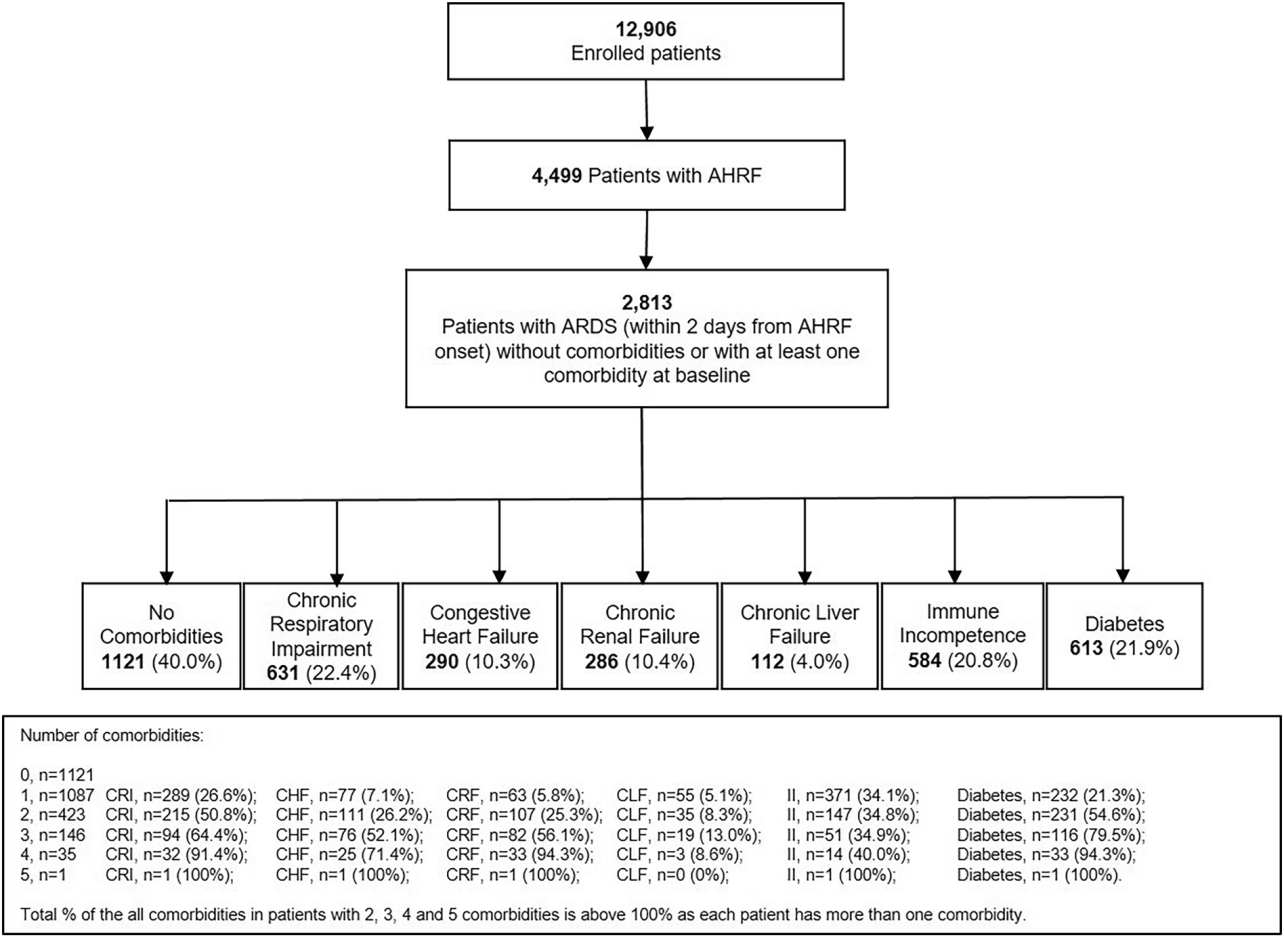
Selection of patients for study population. Patients with more than one comorbidity will be classified on the basis of each comorbidity that they have

Inclusion criteria were admission to a study ICU (including ICU transfers) within the 4-week enrollment window and receipt of invasive or non-invasive ventilation. Exclusion criteria were age < 16 years or inability to obtain informed consent (where required). Patients were classified as having ARDS based on whether or not they fulfilled all of the Berlin criteria rather than by clinician determination, as previously described [6]. We restricted subsequent analyses to patients (93%, *n* = 2813) that fulfilled ARDS criteria within 48 h of the onset of acute hypoxemic respiratory failure (AHRF) (Fig. 1). All data were recorded for each patient at the same time each day within participating ICUs, normally as close as possible to 10am each day. Data on ventilatory settings were recorded simultaneously with arterial blood gas analysis.

### Data definitions

Our data definitions have been previously reported [6–8]. Data collected on comorbidities were the following:

- Chronic respiratory impairment, a patient has known or suspected chronic obstructive pulmonary disease or home ventilation therapy;
- Congestive heart failure, a patient has chronic heart failure with marked limitation of physical activity or is unable to carry out any physical activity without chest discomfort (NYHA Classes III–IV);
- Chronic renal failure, a patient has chronic renal failure with a creatinine clearance less than 60 ml per minute:
- Chronic liver failure, a patient has chronic liver disease with a calculated Child Pugh score ≥ 10;
- Immune incompetence: a patient has (a) a solid tumor which has not been resected or in remission, which is still requiring treatment or with metastasis; (b) viral immunosuppression, neoplastic disease, immunosuppressive drugs (including steroids), chemotherapy or congenital immunosuppression illness; or (c) an active hematologic neoplasm still requiring treatment;
- Diabetes: a patient has known diabetes mellitus treated by drugs or diet.

For the purposes of this analysis, patients with more than one comorbidity appear in each relevant comorbidity category. Duration of invasive mechanical ventilation was calculated as the number of days between the date of intubation and the date of extubation in ICU (or death, if the patient died while receiving invasive mechanical ventilation). Survival was evaluated at ICU discharge or at hospital discharge up to a 90-day follow-up. Data about limitation of life-sustaining measures were reported. We defined new and/or worsening systemic acute organ dysfunction as an increase of ≥ 1 in SOFA score, in patients with an admission score of < 3 for that component of the SOFA score at 28-day follow-up.

### Data management and statistical analyses

Descriptive statistics included proportions for categorical and mean (standard deviation) or median (interquartile range) for continuous variables. The study population was defined at patient cohort that developed ARDS within the first 2 days of developing hypoxic respiratory failure [6, 7, 9]. Comparisons between patients with any comorbidities or a specific type of comorbidities with patients with no comorbidities were performed using Chi-squared test (or Fisher exact test) for discrete variables, Student’s t-test (or Wilcoxon–Mann–Whitney test) for continuous variables. The Shapiro–Wilk test and the visual inspection of the data distribution was used to assess normality.

To evaluate factors associated with outcome from ARDS (i.e., ICU and hospital mortality), we applied multivariable logistic regression model and the independent predictors (demographic characteristics and clinical parameters measured at the first day of AHRF or ARDS) were identified through stepwise regression approach. The level of association was evaluated by OR with 95% confidence interval (90% CI). This approach combines forward and backward selection methods in an iterative procedure to select predictors in the final multivariable model. This approach was also applied to identify factors associated with decisions to limit life-sustaining measures.

In order to test the difference in mortality and limitation of life-sustaining measures among patients with increasing numbers of comorbidities, patients were stratified according to 4 categories (i.e., no comorbidities, a single comorbidity, 2 comorbidities, or 3 or more comorbidities). A Kaplan–Meier analysis was performed to detect differences across these 4 groups 90-day (i.e., ICU and hospital mortality, and limitation of life-sustaining measures) follow-up. Statistical difference between survival curves was assessed by log-rank test. Furthermore, we applied survival analysis with competing risk in order to investigate the relationship between increasing number of comorbidities and likelihood of limitation of life-sustaining treatment during 90-day follow-up, considering ICU death as competing risk (i.e., event that precludes the occurrence of limitation of life-sustaining measures). In this case, Fine and Gray competing risk regression model was used to assess the effect of comorbidities by the estimation of subhazard ratios with 95% confidence interval.

All p-values were two-sided, with *p*-values < 0.05 considered as statistically significant.

Statistical analyses were performed with STATA/16 MP (Texas, USA), GraphPad Prism 8.0.2 (La Jolla, California, USA), R software, version 3.3.3 (R Project for Statistical Computing, http://www.R-project.org) and SAS software, version 9.4 (SAS Institute, Cary, NC, USA).

## Results

Of 4,499 patients that developed acute hypoxic respiratory failure (AHRF), 2,813 patients developed ARDS within the first 2 days of developing AHRF, of which 1692 (60.1%) had at least 1 major comorbidity (Fig. 1, Table 1). Of these, 1087 (64%) had one major comorbidity, 423 (25%) had two major comorbidities, while 182 (11%) had three or more major comorbidities. The order of frequency of comorbid conditions was chronic respiratory impairment (*n* = 631, 22.4%), diabetes (*n* = 613, 21.8%) immune incompetence (*n* = 584, 20.8%), chronic renal failure (*n* = 286, 10.2%), congestive heart failure (*n* = 290, 10.3%), and chronic liver failure (*n* = 112, 4%) (Fig. 1, Table 1).

**Table 1:** Demographics and illness severity profiles of patients with ARDS with and without comorbidities

Parameter (*n* = 2813)^1^	No comorbidities	Any comorbidities*	Chronic respiratory impairment	Congestive heart failure	Chronic renal failure	Chronic liver failure	Immune incompetence	Diabetes
Patients, *n* (%)	1121 (39.9)	1692 (60.1)	631 (22.4)	290 (10.3)	286 (10.2)	112 (4.0)	584 (20.8)	613 (21.8)
Male patients, *n* (%)	687 (61.3)	1042 (61.6)	423 (67.0)*	182 (62.8)	190 (66.4)	77 (68.8)	337 (57.7)	388 (63.3)
Age (years), mean ± SD	56 ± 18	65 ± 15*	68 ± 13*	72 ± 12*	69 ± 13*	56 ± 11	60 ± 16*	67 ± 13*
BMI (kg/m^2^), median (IQR)	25.8 (23.0–29.7)	26.1 (22.8–30.9)	25.9 (22.9–30.1)	26.3 (22.9–32.1)	27.3 (24.1–32.4)*	26.7 (22.8–30.6)	24.9 (21.6–28.4)*	28.1 (24.2–34.2)*
Geo-economic areas, *n* (%)								
High-income Europe	628 (56.0)	893 (52.8)	357 (56.6)	159 (54.8)	140 (49.0)*	54 (48.2)	315 (54.0)	293 (47.8)*
High-income RW	270 (24.1)	476 (28.1)	165 (26.1)	62 (21.4)	99 (34.6)*	42 (37.5)*	176 (30.1)*	209 (34.1)*
Middle income	223 (19.9)	323 (19.1)	109 (17.3)	69 (23.8)	47 (16.4)	16 (14.3)	93 (15.9)*	111 (18.1)
Clinician recognition of ARDS, n (%)								
At baseline	373 (33.3)	514 (30.4)	141 (22.3)*	70 (24.1)*	82 (28.7)	38 (33.9)	217 (37.2)	188 (30.7)
At any time	693 (61.8)	1042 (61.6)	345 (54.7)*	154 (53.1)*	166 (58.0)	75 (70.0)	410 (70.2)*	375 (61.2)
No longer fulfill ARDS criteria after 24 h, n (%)	195 (17.4)	291 (17.2)	111 (17.6)	58 (20.0)	59 (20.6)	19 (17.0)	94 (16.1)	98 (16.0)
ARDS Severity, *n* (%)								
Mild ARDS	326 (29.1)	507 (30.0)	186 (29.5)	96 (33.1)	96 (33.6)	35 (31.2)	167 (28.6)	181 (29.5)
Moderate ARDS	516 (46.0)	822 (48.6)	323 (51.2)*	139 (47.9)	142 (49.6)	47 (42.0)	271 (46.4)	305 (49.8)
Severe ARDS	279 (24.9)	363 (21.4)*	122 (19.3)*	55 (19.0)*	48 (16.8)*	30 (26.8)	146 (25.0)	127 (20.7)
PaO_2_/FiO_2_ (mmHg) at day 1 of ARDS, mean ± SD	157 ± 68	162 ± 67	162 ± 64	167 ± 67*	172 ± 68*	161 ± 71	157 ± 68	163 ± 67
pCO_2_ (mmHg), at day 1 of ARDS, mean ± SD	44 ± 14	47 ± 16*	54 ± 18*	49 ± 17*	44 ± 15	41 ± 12*	44 ± 16	46 ± 16
pH, at day 1 of ARDS, mean ± SD	7.34 ± 0.12	7.33 ± 0.12*	7.32 ± 0.12*	7.33 ± 0.11	7.33 ± 0.12	7.31 ± 0.12*	7.34 ± 0.12	7.33 ± 0.13
Total SOFA adjusted Score, mean ± SD								
Day 1 of AHRF	9.3 ± 3.9	9.4 ± 4.2	8.6 ± 4.0*	9.3 ± 3.9	10.3 ± 3.6*	13.7 ± 4.1*	9.4 ± 3.9	9.5 ± 4.1
Worst SOFA within 28 days in ICU	10.9 ± 4.2	11.2 ± 4.4	10.2 ± 4.4*	11.1 ± 4.3	11.9 ± 3.9*	15.2 ± 3.9*	11.5 ± 4.3*	11.2 ± 4.4
Non-pulmonary SOFA adjusted score, mean ± SD								
Day 1 of AHRF	6.0 ± 3.9	6.3 ± 4.1	5.4 ± 4.0*	6.2 ± 3.9	7.2 ± 3.7*	10.6 ± 4.0*	6.3 ± 4.0	6.3 ± 4.1
Worst SOFA within 28 days in ICU	7.7 ± 4.0	8.1 ± 4.3*	7.1 ± 4.3*	8.1 ± 4.2	8.9 ± 3.9*	12.0 ± 3.7*	8.4 ± 4.2*	8.2 ± 4.3*
Type of admission, *n* (%)								
Medical	783 (69.9)	1375 (81.3)*	519 (82.3)*	231 (79.7)*	239 (83.6)*	94 (83.9)*	474 (81.2)*	484 (79.0)*
Post-operative	50 (4.5)	103 (6.1)	36 (5.7)	23 (7.9)*	15 (5.2)	10 (8.9)*	40 (6.9)*	36 (5.9)
Surgical	200 (17.8)	193 (11.4)*	66 (10.5)*	32 (11.3)*	29 (10.1)*	6 (5.4)*	69 (11.8)*	80 (13.1)*
Trauma	88 (7.9)	21 (1.2)*	10 (1.6)*	4 (1.4)*	3 (1.0)*	2 (1.8)*	1 (0.2)*	13 (2.1)*
Number of comorbidities, *n* (%)								
0	1121 (100)	0 (0)	0 (0)	0 (0)	0 (0)	0 (0)	0 (0)	0 (0)
1	–	1087 (64)	289 (46)	77 (27)	63 (22)	55 (49)	371 (64)	232 (38)
2	–	423 (25)	215 (34)	111 (38)	107 (37)	35 (31)	147 (25)	231 (38)
≥ 3	–	182 (11)	127 (20)	102 (35)	116 (41)	22 (20)	66 (11)	150 (24)

Test used for continuous variables: Wilcoxon rank sum (all variables are not normally distributed); for categorical variables: Chi square. *The group “Any comorbidities” includes less patients than the sum of the single comorbidities as patients could have more than 1 comorbidity

^*^ < 0.05 versus no comorbidities

### Patient demographics

Patient with no comorbidities were younger than patients with comorbidities, except for those with chronic liver failure. Patients with chronic renal failure and those with diabetes had higher BMI than patients with no comorbidities (Table 1). There was a higher frequency of chronic renal failure, chronic liver failure, and diabetes in patients from high-income countries outside Europe, compared to European high-income country patients or those from middle-income countries (Additional file 1: Table S1). ARDS was less likely to be recognized in patients with chronic respiratory impairment and congestive heart failure or immune incompetence, compared to those with no comorbidities (Table 1). A medical reason for admission was more commonly present in patients with any type of comorbidities compared to those with no comorbidities (Table 1). Additional data regarding the patient population are reported in Additional file 1: Table S2.

### Illness severity profiles

Patient with no comorbidities had a higher rate of severe ARDS compared to patients with chronic respiratory impairment, CHF and chronic renal failure. SOFA scores were lower in patients with chronic respiratory impairment, and higher in patients with renal or hepatic failure and immune incompetence, compared to those with no comorbidities. Non-pulmonary SOFA scores were lower in patients with chronic respiratory impairment, and higher in patients with chronic renal failure, chronic liver failure and immune incompetence.

### Management of ARDS

The use of invasive ventilation (*n* = 1379 versus *n* = 998, 82 versus 89%), neuromuscular blockade (*n* = 301 versus *n* = 249, 18 versus 22%), prone positioning (*n* = 97 versus *n* = 104, 6 versus 9%) and ECMO (*n* = 32 versus *n* = 46, 2 versus 4%) were each significantly reduced in patients with comorbidities. More patients with chronic respiratory impairment, CHF, chronic renal failure, immune incompetence and diabetes received non-invasive ventilation, while more patients with chronic liver failure received invasive MV, compared to patients with no comorbidities (Additional file 1: Table S3). Overall, the use of adjunctive strategies was less frequent in patients with comorbidities. Fewer patients with chronic respiratory impairment and with CHF, renal and liver failure, and diabetes received continuous neuromuscular blockade. A similar pattern was seen for prone position ventilation (Additional file 1: Table S3). Factors independently associated with the use of any adjunctive measure within 28-day follow-up, included invasive ventilation, clinical recognition at baseline, hypoxemia and higher ventilatory pressures (i.e., PIP and PEEP). Among the comorbidities, immune incompetence and diabetes were independently correlated with the use of adjunctive measures within 28-day follow-up (Table 2).

**Table 2:** Multivariate logistic regression model of factors associated with the use of any adjunctive measures within 28-day follow-up

Variable	OR	95% CI	p
Age	0.99	0.98–1.00	0.001
Comorbidity			
Chronic respiratory impairment	0.99	0.79–1.24	0.916
Congestive heart failure	1.20	0.89–1.62	0.241
Chronic renal failure	1.01	0.75–1.38	0.927
Chronic liver failure	0.65	0.41–1.03	0.069
Immune incompetence	1.27	1.02–1.57	0.030
Diabetes	1.28	1.03–1.57	0.027
Clinical recognition at baseline	1.66	1.38–2.00	< 0.001
Invasive mechanical ventilation (Ref. No.)	1.86	1.37–2.52	< 0.001
PaO_2_/FiO_2_ (for each 10 mmHg)	0.95	0.93–0.96	< 0.001
PIP	1.03	1.02–1.04	< 0.001
PEEP	1.15	1.12–1.19	< 0.001
High-income RW (vs Europe)	0.40	0.32–0.50	< 0.001
Middle-income countries (vs Europe)	0.78	0.62–0.98	0.030

Sample size, *n* = 2654

*BMI* body mass index, *SOFA* Sequential Organ Failure Assessment, *PEEP* positive end-expiratory pressure, *PIP* peak inspiratory pressure, *RR* respiratory rate, *RW* rest of the world

### Outcomes from ARDS

There were no major differences in the rates development of new or worsening systemic organ dysfunction in ARDS patients with comorbidities compared to those with no comorbidities (Additional file 1: Table S4). Similar proportions of patients with and without comorbidities no longer fulfilled ARDS criteria after 24 h. The number of MV-free days was higher in patients with no comorbidity compared to patients with any of the studied comorbidity except in patients with chronic respiratory impairment. Similar findings were observed regarding ICU and hospital length of stay (Table 3).

**Table 3:** Outcomes of patients with ARDS with and without comorbidities

Parameter	No comorbidities (*n* = 1121)	Any comorbidities (*n* = 1692)*	Chronic respiratory impairment (*n* = 631)	Congestive heart failure (*n* = 290)	Chronic renal failure (*n* = 286)	Chronic liver failure (*n* = 112)	Immune incompetence (*n* = 584)	Diabetes (*n* = 613)
Invasive ventilation-free days, median (IQR), days								
All patients, *N* = 2377	15 (0–23)	5 (0–23)*	15 (0–23)	4 (0–22)*	0 (0–23)*	0 (0–19)*	0 (0–22)*	10 (0–23)*
Survivors, *N* = 1539	20 (13–25)	21 (16–25)*	22 (16–25)*	22 (16–25)	22 (12–25)	22 (20–25)	22 (16–25)	21 (15–25)
Duration of invasive mechanical ventilation, median (IQR), days								
All patients, *N* = 2377	8 (4–16)	8 (4–14)	7 (4–14)	8 (3–14)	8 (4–18)	7 (3–12)*	8 (4–14)	8 (4–15)
Surviving patients, *N* = 1539	9 (4–16)	8 (4–14)*	7 (4–13)*	8 (4–13)	7 (4–17)	7 (4–9)	7 (4–13)	8 (4–14)
ARDS criteria, n (%)								
Still present 24 h after diagnosis *N* = 1611	683 (76.1)	928 (76.3)	334 (75.4)	147 (73.9)	147 (74.2)	64 (76.2)	327 (77.9)	346 (76.5)
Resolved ARDS, *N* = 503	215 (23.9)	288 (23.7)	109 (24.6)	52 (26.1)	51 (25.8)	20 (23.8)	93 (22.1)	106 (23.5)
Duration of ICU Stay, median (IQR), days								
All patients, *N* = 2813	11 (5–20)	9 (5–17)*	9 (4–16)*	9 (4–17)*	9 (5–20)	8 (4–15)*	10 (5–17)*	10 (5–18)
Survivors, *N* = 1849	12 (6–21)	10 (6–18)*	9 (5–17)*	10 (5–18)*	9 (6–19)*	10 (6–16)	10 (6–17)*	10 (6–19)
Duration of hospital stay, median (IQR), days								
All patients, *N* = 2813	18 (9–35)	16 (8–29)*	16 (8–27)*	14 (7–25)*	16 (8–28)*	10 (4–23)*	17 (8–29)*	16 (9–31)
Survivors, *N* = 1695	23 (13–40)	22 (12–38)	19 (11–33)*	20 (11–35)*	22 (13–43)	26 (16–56)	24 (15–40)	21 (13–40)
ICU mortality, No. (%)	303 (27.0)	661 (39.1)*	209 (33.1)*	120 (41.4)*	113 (39.5)*	75 (67.0)*	266 (45.5)*	210 (34.3)*
Hospital mortality, No. (%)	347 (31.0)	762 (45.3)*	242 (38.7)*	135 (46.9)*	138 (48.4)*	81 (72.3)*	304 (52.4)*	254 (41.6)*
Limitation of life-sustaining measures, n (%)	209 (18.6)	493 (29.1)*	162 (25.7)*	87 (30.0)*	83 (29.0)*	49 (43.8)*	195 (33.4)*	158 (25.8)
Withhold	165 (15.4)	408 (25.3)*	131 (21.6)*	70 (24.9)*	65 (23.9)*	43 (39.8)*	158 (28.6)*	142 (24.2)*
Withdraw	336 (20.9)	149 (13.9)*	113 (18.8)*	57 (20.7)*	64 (23.3)*	39 (35.8)*	129 (23.6)*	107 (18.2)*

Test used for continuous variables: Wilcoxon rank sum (all variables are not normally distributed); for categorical variables: Chi square. Missing information on hospital mortality in 9 patients. *The group “Any comorbidities” includes less patients than the sum of the single comorbidities as patients could have more than 1 comorbidity

^*^ < 0.05 versus No comorbidities

ICU mortality increased from 27% (*n* = 303) in patients with no comorbidity to 39% (*n* = 661) in patients with any comorbidity. ICU mortality was lowest in patients with no comorbidity (*n* = 303, 27%) and increased progressively in patients with chronic respiratory impairment (*n* = 209, 33%), diabetes (*n* = 210, 34%), chronic renal failure (*n* = 113, 40%), chronic heart failure (*n* = 120, 41%), immune incompetence (*n* = 266, 46%), and chronic liver failure (*n* = 75, 67%). A similar pattern was seen regarding hospital mortality, where mortality increased from 31% (n = 347) in patients with no comorbidity to 72% (*n* = 81) in patients with chronic liver failure (Table 3).

**Fig. 2:**
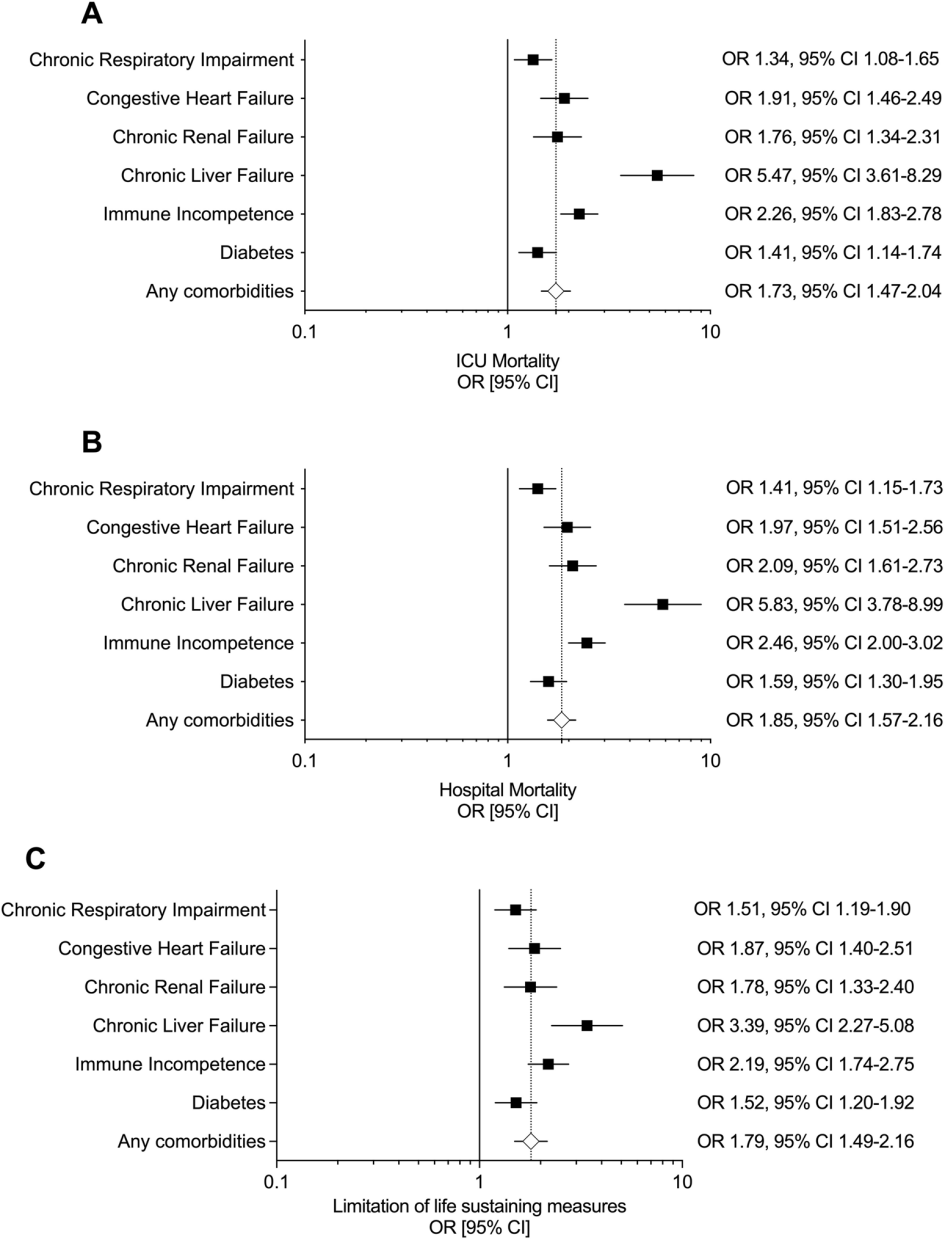
**A** ICU mortality, **B** hospital mortality and **C** limitation of life-sustaining measures as a function of patients with comorbidities. Unadjusted odds ratio calculated versus patients with no comorbidities

At the univariate analysis, all the studied comorbidities were significantly associated with a higher risk of ICU (Fig. 2 panel A) and hospital mortality at 90 days (Fig. 2 panel B). The risk of ICU and hospital mortality stratified by the type of immune incompetence is reported in Additional file 1: Fig. S1, panel A and B.

After adjusting these findings for multiple confounders, we observed that CHF, chronic liver failure and immune incompetence remained independent predictors of ICU mortality among all the comorbidities (Table 4). Chronic liver failure and immune incompetence, but not CHF, were predictive of hospital mortality at 90-day follow-up (Additional file 1: Table S5).

**Table 4:** Multivariate logistic regression model of factors associated with the ICU mortality in all patients

Variable	OR	95% CI	p
Age	1.02	1.01–1.03	< 0.001
BMI	0.98	0.97–1.00	0.022
Comorbidity			
Chronic respiratory impairment	0.98	0.78–1.23	0.839
Congestive heart failure	1.42	1.07–1.90	0.016
Chronic renal failure	0.97	0.72–1.31	0.858
Chronic liver failure	3.35	2.11–5.34	< 0.001
Immune incompetence	1.96	1.58–2.42	< 0.001
Diabetes	0.91	0.73–1.15	0.440
Medical admission	1.51	1.21–1.89	< 0.001
No longer fulfill ARDS criteria after 24 h	0.72	0.56–0.92	0.010
Adjusted non-respiratory SOFA	1.11	1.08–1.14	< 0.001
pH	0.74	0.67–0.81	< 0.001
PaO_2_/FiO_2_	0.98	0.97–1.00	0.013
paCO_2_	0.99	0.98–1.00	0.019
PEEP	0.96	0.93–0.99	0.005
Total respiratory rate	1.03	1.02–1.04	< 0.001
No use of adjunctive measures within 28-day follow-up (Ref. Use of adjuncts)	0.64	0.52–0.77	< 0.001
High-income RW (vs Europe)	0.71	0.57–0.89	0.003
Middle-income countries (vs Europe)	1.62	1.29–2.04	< 0.001

Sample size *n* = 2611

*BMI* body mass index, *SOFA* Sequential Organ Failure Assessment, *PEEP* positive end-expiratory pressure, *RR* respiratory rate, *RW* rest of the world

**Fig. 3:**
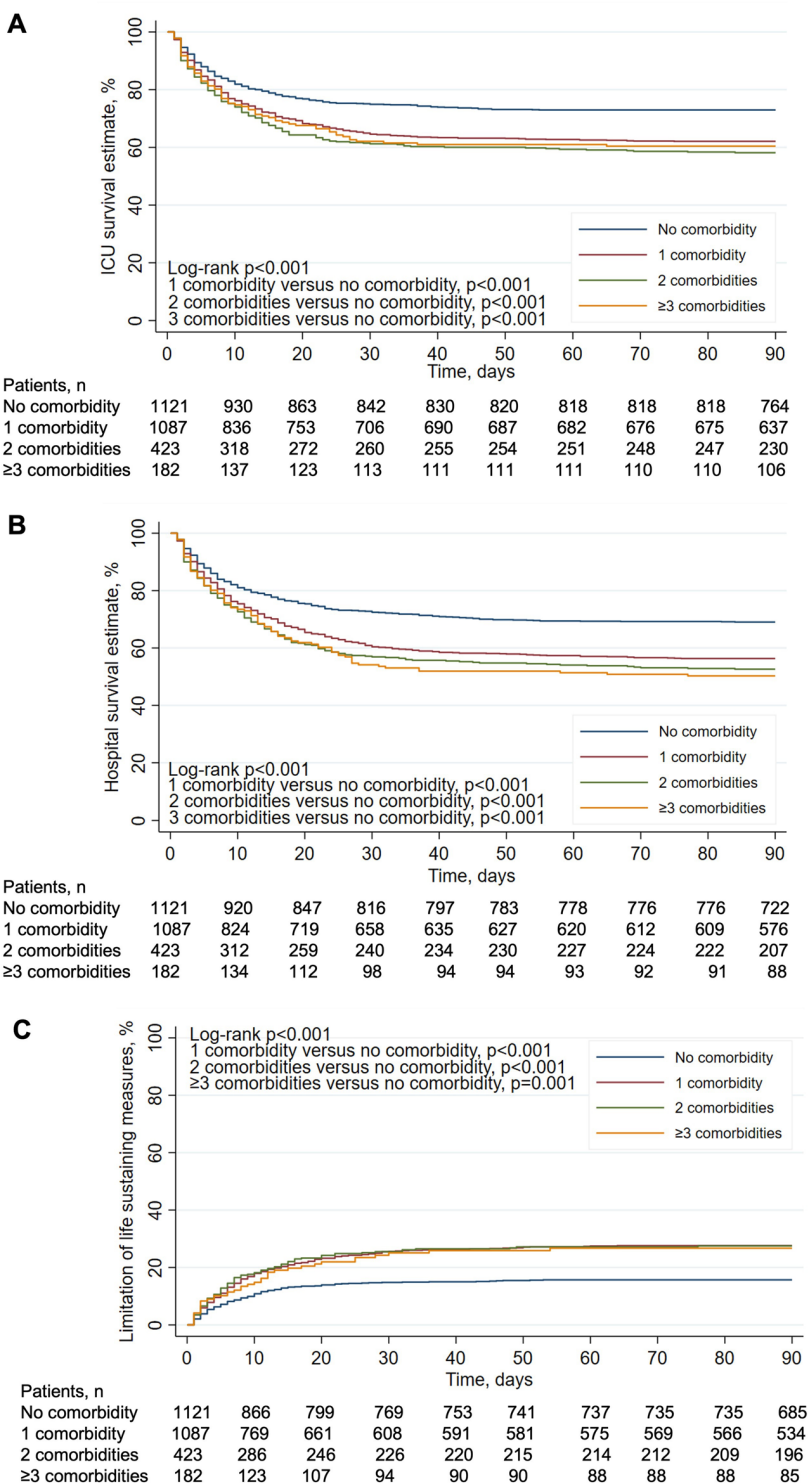
Unadjusted probability of ICU (**A**) and hospital survival (**B**) and limitation of life-sustaining measures (**C**) at 90-day follow-up by increasing number of comorbidities. We used the log-rank test to assess the differences between curves

Furthermore, the presence of 1, 2, or > 3 comorbidities was significantly associated with a lower ICU (*n* = 675, *n* = 246, *n* = 110 versus no comorbidity—*n* = 818; 62%, 58%, 60% versus 73%, log-rank *p* < 0.001) (Fig. 3, panel A) and hospital survival (*n* = 609, *n* = 221, *n* = 91 versus no comorbidity—*n* = 774; 56%, 53%, 50% versus 69%, log-rank *p* < 0.001) (Fig. 3, panel B) at 90-day follow-up.

Decisions to limit life-sustaining measures were more frequent in patients with any comorbidities compared to those without comorbidities (*n* = 493 versus *n* = 209; 29% versus 19%, *p* < 0.001). Differences in limitation of care remained significant when patients were stratified by single groups of comorbidities (Table 3).

At univariate analysis, all the studied comorbidities were significantly associated with a higher proportion of limitation of life-sustaining measures (Fig. 2 panel C). The risk of a decision to limit life-sustaining measures by the type of immune incompetence is reported in Additional file 1: Fig. S1, panel C.

After adjusting for multiple confounders, both chronic liver failure and immune incompetence were predictive of limitation of life-sustaining measures at 90-day follow-up (Table 5). Furthermore, the presence of 1, 2, or > 3 comorbidities were each significantly associated with an increase in the proportion of limitation of life-sustaining measures (*n* = 315, *n* = 124, *n* = 54 versus no comorbidity—*n* = 208; 29%, 29%, 30% versus 19%, log-rank *p* < 0.001) (Fig. 3, panel C) at 90-day follow-up. Similar findings were obtained considering ICU mortality as a competing risk that precludes the occurrence of the event of interest (i.e., limitation of life-sustaining measures) (Additional file 1: Fig. S2).

**Table 5:** Multivariate logistic regression model of factors associated with limitation of life-sustaining measures

Variable	OR	95% CI	p
Age	1.03	1.02–1.03	< 0.001
BMI	0.98	0.96–0.99	< 0.001
Comorbidity			
Chronic respiratory impairment	0.94	0.74–1.19	0.604
Congestive heart failure	1.25	0.92–1.70	0.156
Chronic renal failure	1.00	0.73–1.37	0.988
Chronic liver failure	2.22	1.40–3.53	0.001
Immune incompetence	1.58	1.25–1.99	< 0.001
Diabetes	0.97	0.76–1.24	0.819
Medical admission	1.37	1.07–1.76	0.013
Adjusted non-respiratory SOFA	1.05	1.03–1.08	< 0.001
pH	0.88	0.80–0.95	0.002
PEEP	0.95	0.92–0.98	0.004
Total RR	1.04	1.02–1.05	< 0.001
High-income RW (vs Europe)	0.72	0.55–0.94	0.016
Middle-income countries (vs Europe)	0.61	0.45–0.81	0.001
Number of beds per physician	1.03	1.01–1.06	0.001

Sample size, *n* = 2391

*BMI* body mass index, *SOFA* Sequential Organ Failure Assessment, *PEEP* positive end-expiratory pressure, *RR* respiratory rate, *RW* rest of the world

With regard to the organ system failure considered by clinicians most responsible for death, cardiovascular failure was more frequent in patients with CHF and less frequent in patients with chronic liver failure—who died more of hepatic failure. Respiratory failure was more common in patients with immune incompetence, compared to patients with no comorbidities (Additional file 1: Table S6).

## Discussion

The frequency and impact of underlying comorbidities on the clinical presentation, management and outcomes in patients with ARDS remain incompletely understood. We report that in the LUNG SAFE patient cohort, a large and geographically diverse cohort of ‘real world’ patient with ARDS, most patients had at least one significant comorbidity. We further found that patients with comorbidities were managed differently, being less likely to receive higher intensity modalities of organ support and adjunctive therapies. Patients with comorbidities experienced differences in end-of-life care and were more likely to die in the ICU and in hospital. These findings raise concerns regarding the management and outcomes of these patients and suggest that enhancing the care of these patients must be a priority for future clinical studies.

Critically ill ARDS patients with comorbidities were older and were more frequently admitted with acute medical condition. Geo-economic differences existed in comorbidity profiles, with a higher frequency of chronic renal failure, chronic liver failure, immune incompetence and diabetes in patients from high-income countries outside Europe. Of interest, ARDS was less likely to be recognized in patients with comorbidities, especially chronic respiratory impairment, congestive heart failure or immune incompetence. Overall, patients with comorbidities had less severe ARDS, and there were potentially important differences in the pattern of extrapulmonary organ injury.

The presence of comorbidities in our study population had a major impact on the management of ARDS patients. Patients with underlying comorbidities were managed more frequently with non-invasive ventilation, they received less adjunctive measures including NMBs, prone positioning and ECMO, regardless of their level of evidence [10], raising the concern of a potential bias towards less aggressive treatment of patients with comorbidities. It should be acknowledged that patients with comorbidities may have more contra-indications to the use of these management strategies, and/or the treating clinician may consider that these approaches may not be suitable for specific patients. Factors such as clinician recognition of ARDS at baseline, the use of invasive mechanical ventilation, greater ARDS severity (i.e., a lower PF ratio), a higher peak inspiratory and positive end-expiratory pressure were all independently associated with the use of adjunctive measures within 28-day follow-up. Furthermore, among all comorbidities immune incompetence and diabetes were independent predictors of using ARDS adjuncts during ICU stay.

The fact that ARDS patients with significant underling comorbidities are frequently excluded from interventional clinical trials, particularly trials of novel investigative medicinal products, has raised concerns regarding the generalizability of the findings of these studies. These concerns were first raised by Azoulay et al., in their study of the impact of comorbidities in a French cohort of patients with ARDS [11], where 51% of their cohort had significant comorbidities. Our findings confirm and extend their findings, demonstrating that the majority of patients in this global ARDS cohort had comorbidities that may have led to their being excluded from a clinical trial of an intervention for ARDS. Furthermore, clinician concerns regarding the generalizability of findings from ARDS trials of novel therapeutics to patients with underlying comorbidities may contribute to the differences seen in patient management.

The differences between cohorts of ARDS patients with and without comorbidities, in terms of their demographic profile, and the clinical pattern of ARDS, raises the possibility that patients with significant comorbidities may respond differently to these management strategies. These concerns are underlined by the findings that patients with different ARDS subphenotypes appear to respond differently to PEEP and to statin therapy [12, 13], while patients with different patterns of ARDS may respond differently to ventilation strategies [14]. These issues underline the need for additional studies focused on this patient population to develop the evidence base for their management.

ARDS patients with comorbidities had worse outcomes than those with no comorbidities. Specifically, these patients had less ventilator-free days, while ICU and hospital mortality was higher in this cohort. ICU survival in patients with no comorbidities at 73% compares favorably with outcomes from recent clinical trials [15, 16] and provides a ‘real world’ benchmark for outcomes in control populations in studies focused on this cohort. In contrast, ICU survival decreased to 61% in patients with at least 1 significant comorbidity.

The presence of even 1 major comorbidity increased the risk of death, there further increase in the risk of death with a greater number of comorbidities present. Specific comorbidities, including congestive heart failure, chronic liver failure and immune incompetence were each independent predictors of ICU mortality, while both chronic liver failure and immune incompetence were independently associated with hospital mortality. The presence of 1 or more comorbidities was associated with a higher proportion of limitation of life-sustaining measures compared to patients without comorbidities. Both chronic liver failure and immune incompetence were both independently associated with more decisions regarding end-of-life care. Of interest, we observed that a higher number of beds per physician (i.e., an index of resource ‘strain’) and location in a high-income European ICU were both positive predictors of a higher decision to limit treatment.

## Limitations

This study has several limitations. Our patient cohort, while large and geographically diverse, may not be representative of actual clinical practice in ICUs across the globe. We did not have access to the source data for the patients in the enrolling ICUs, and it is possible that not all patients with ARDS in participating centers were enrolled. However, enrollment of patients with ARDS from participating ICUs met expectations based on their recorded 2013 admission rates, while data from lower recruiting ICUs were not different from that from higher enrolling ICUs, suggesting the absence of reporting biases. We instituted a robust data quality control program in which all centers were requested to verify data that appeared inconsistent or erroneous. While we have adjusted our analyses to account for known measured confounders, the possibility remains that some of our findings may arise from unmeasured or residual confounding. Moreover, we cannot make causal inferences for the associations seen, given the observational nature of our study. It should be acknowledged that the LUNG SAFE study unveiled that a significant proportion of patients did not receive protective mechanical ventilation, PEEP levels stratified by ARDS severity [6] and widely available adjuncts such as neuromuscular blockade and prone position [10] despite guidelines recommendations [17, 18]. This may imply that the relationship between comorbidities and outcome (i.e., mortality and limitation of life-sustaining measures) may differ as compared to cohort of ARDS patients treated according to the ARDS guidelines. The lack of information regarding the use of non-pulmonary organ supports should be considered as a limitation of the study.

Furthermore, as the patient inclusion period was before the pandemic era, the findings of the present study may not apply to patients with COVID-19 ARDS.

## Conclusions

Our findings demonstrate that 60% of patients with ARDS have 1 or more significant comorbidities**.** These comorbidities profoundly influence patient management, with these patients less likely to receive invasive ventilation, neuromuscular blockade, prone positioning or ECMO. These findings raise concerns regarding both the use, and the applicability, of current management strategies in these patients. The impact on patient outcome is clear, with patients with comorbidities being more likely to receive treatment limitation decisions, while ICU mortality is over 40% higher than in patients without these comorbidities. Advancing the care of these patients must be a priority for future clinical studies.

## Supplementary Information

**Additional file 1:** **Figure S1. **A) ICU mortality, B) Hospital mortality and C) Limitation of care as a function of patients with different comorbidities that characterize the immune incompetence (i.e., solid neoplasm, hematological malignancy and immune-suppression). Unadjusted odds ratio calculated versus patients with no comorbidities. **Figure S2.** Survival analysis with competing risk to investigate the relationship between increasing number of comorbidities and the likelihood of limitation of life-sustaining measures during 90-day follow-up, considering ICU death as competing risk (i.e., event that precludes the occurrence of limitation of life-sustaining measures). **Table S1.** Comorbidities stratified by major geoeconomic areas of patients with ARDS. **Table S2.** ARDS risk factors, comorbidity profile and ICU related variables of patients with ARDS with and without comorbidities. **Table S3.** Management of patients with ARDS with and without comorbidities. **Table S4.** Development of new and/or worsening systemic acute organ dysfunction† in the study population. **Table S5.** Multivariate logistic regression model of factors associated with the hospital mortality in all patients. **Table S6.** Organ system failure considered as most important factors leading to death in ICU in non-surviving patients with ARDS at 90-day follow-up. **Appendix S1.**

## Data Availability

The datasets used and/or analyzed during the current study are available from the corresponding author on reasonable request.

## Abbreviations

AHRF: Hypoxemic respiratory failure
ARDS: Acute respiratory distress syndrome
CHF: Congestive heart failure
CRF: Chronic renal failure
ECMO: Extracorporeal membrane oxygenation
ICU: Intensive Care Units
MV: Mechanical ventilation
NMBA: Neuromuscular blocking agent
PEEP: Positive end-expiratory pressure
PF ratio: PaO_2_/FiO_2_
PEEP: Positive end-expiratory pressure
PP: Prone positioning
SOFA: Sequential Organ Failure Assessment
